# Identification of a Novel Dehydroergosterol Enhancing Microglial Anti-Inflammatory Activity in a Dairy Product Fermented with *Penicillium candidum*

**DOI:** 10.1371/journal.pone.0116598

**Published:** 2015-03-11

**Authors:** Yasuhisa Ano, Toshiko Kutsukake, Ayaka Hoshi, Aruto Yoshida, Hiroyuki Nakayama

**Affiliations:** 1 Central Laboratories for Key Technologies, Kirin Company Ltd, 1–13–5 Fukuura Kanazawa-ku, Yokohama-shi, Kanagawa, 236–0004, Japan; 2 Graduate School of Agricultural and Life Sciences, the University of Tokyo, 1–1–1 Yayoi, Bunkyo-ku, Tokyo, 113–8657, Japan; University of Cologne, GERMANY

## Abstract

Despite the ever-increasing number of dementia patients worldwide, fundamental therapeutic approaches to treat this disease remain to be established. Preventive approaches such as diet, exercise and learning attract attention. Several epidemiological studies suggest that ingestion of fermented dairy products prevents cognitive decline in the elderly. These reports indicate that specific ingredients in the fermented dairy products elicit an anti-inflammatory or anti-oxidative activity that facilitates neuroprotection. The responsible components remain to be investigated. A number of studies have shown that inflammation caused by microglia is closely related to exaggeration of the pathology and cognitive decline seen in the elderly. Many researchers have proposed that controlling microglial activities could be effective in preventing and possibly curing dementia. In the present study, to elucidate specific compounds that regulate microglial activity from dairy products, repeated purification by HPLC, combined with evaluation using primary microglia, facilitated the identification of dehydroergosterol (DHE) as a novel component of the extract that enhances microglial anti-inflammatory activity. DHE contains three conjugated double bonds in a steroid ring system and is an analogue of ergosterol. Despite their related chemical structures, the anti-inflammatory activity of DHE is markedly stronger than that of ergosterol. *P*. *candidum* for camembert cheese produces DHE, but *P*. *Roqueforti* for blue cheese and *Aspergillus* do not. DHE also induces CD11b-positive microglia cells into CD206-positive M2 type microglia. Neurotoxicity and neuronal cell death induced by excessively activated microglia is suppressed by treatment with DHE. Thus, this is the first report to demonstrate that DHE, identified as a responsible compound in dairy products, can induce microglia into a preferable phenotype for our brain environment and can be safely introduced into the body by consumption of dairy products. We believe the uptake of DHE might help to prevent the onset of dementia.

## Introduction

Microglia play a central immunological role in nerve tissue by protecting neurons against foreign substances and viruses, as well as helping to remove waste products including apoptotic cells and amyloid proteins *via* phagocytosis [1], [2]. It is recently reported that microglia have a crucial role in terms of neurogenesis and extension of neuronal synapses [3], [4]. As such, microglia are essential for maintaining the brain environment. However, in neurodegenerative disorders, such as Alzheimer’s disease, microglia become excessively activated, accumulating misfolded proteins such as amyloid-β (Aβ) and producing huge quantities of inflammatory cytokines and chemokines such as tumor necrosis factor-α (TNF-α) and macrophage inflammatory protein-1α (MIP-1α), reactive oxygen (ROS) and nitric oxide (NO) [5–7]. These products are chronically toxic for neurons, leading to neuronal cell death and decline in recognition [8], [9].

As a consequence, regulation of the chronic inflammation of microglial cells is essential for maintaining the environment in nervous tissues and preventing the onset of neurodegenerative diseases. Many researcher groups have proposed that controlling microglial activities could be effective in preventing and possibly curing Alzheimer’s diseases and reversing cognitive decline [10–12]. Epidemiological studies suggest that prolonged use of nonsteroidal anti-inflammatory drugs (NSAIDs) significantly reduces the risk of Alzheimer’s disease [13], [14]. Consistent with the epidemiological research, chronic ibuprofen treatment significantly suppressed microglial inflammation and the development of Aβ pathology in the transgenic model mouse for Alzheimer’s disease [15]. However, the side effects of NSAIDs on the gastrointestinal tract, liver and kidney caused by inhibition of cyclooxygenase I preclude their widespread use for the prevention of neurological diseases. Therefore, alternative treatments for Alzheimer’s disease and cognitive decline need to be investigated and preventive approaches *via* changes in dietary regimens have attracted considerable attention.

Recent epidemiological studies suggest that an intake of certain dairy products may reduce the risk of cognitive decline in the elderly and prevent Alzheimer’s disease [16–18]. Camfield et al suggested that specific ingredients, such as peptides and vitamins, are beneficial to promoting healthy brain function during aging. Some reports suggest that dairy products have a positive effect on glucose regulation [16]. Crichton et al revealed that people taking low fat dairy products such as yogurt and cheese once a week have a higher cognitive function than those who are not. As a result of questionnaire surveys and self-reported health information from more than 1,000 participants, consumption of low fat dairy products was found to be associated with increased memory recall, increased social functioning and decreased stress [17]. Ozawa et al surveyed more than 1000 Japanese subjects free from dementia living within the community, aged 60–79 years old, to investigate their dietary patterns and any potential association with reduced risk of dementia [18]. This study concluded that inclusion of milk or fermented dairy products in the diet reduces the risk of dementia in the general Japanese population. Taken together, these reports suggest that specific ingredients in dairy products have an anti-inflammatory or anti-oxidative effect in the brain that elicits neuroprotection. However, the identity of the active ingredients responsible for the beneficial effect on brain function remains unclear.

In this study, we aimed to elucidate specific compounds that strongly suppress microglial inflammation by screening dairy products fermented with *Penicillium candidum*.

## Materials and Methods

### Animals

Pregnant C57BL/6J mice were purchased from Charles River Japan (Tokyo, Japan) and bred in accordance with the procedures authorized by the Animal Experiment Committee of Kirin Company. New born mice under 10-day-old were used for the experiments. Pregnant C57BL/6J mice were maintained in Kirin Company Ltd, and the experiments were approved by the Animal Experiment Committee of Kirin Company and conducted in accordance with their guidelines. All efforts were made to minimize suffering.

### Primary microglia cell culture

To isolate primary microglial cells, mice were euthanized by cervical spine fracture dislocation and removed brains were dissociated with enzyme (Neural Dissociation Kit (P), Miltenyi Biotec, Auburn, CA). Brain cells were treated with anti-CD11b antibody conjugated with microbeads (Miltenyi Biotec) and CD11b-positive cells were isolated by the magnetic cell sorting (MACS) method. Isolated CD11b-positive cells (>90% pure as evaluated by flow cytometer) were plated in a poly-D-lysine (PDL)-coated 96 well plate (BD Biosciences, Billerica, MA) and cultured in DMEM/F-12 (Gibco, Carlsbad, CA) medium supplemented with 10% fetal calf serum (Gibco) and 100 U/ml penicillium/streptomycin (Sigma-Aldrich, St Louis, MO).

### *In vitro* cytokine production assay

Microglia isolated from newborn mice were plated at a density of 30,000 cells per well using PDL-coated plates and then treated with extracted sample as described in the following section, ergosterol (Sigma-Aldrich) or ergosta-5,7,9(11),22-tetren-3β-ol (dehydroergosterol, DHE; Sigma-Aldrich) for 12 hours. Cells were maturated by addition of lipopolysaccharide (LPS, 5 ng/ml, Sigma-Aldrich) and interferon-γ (IFN-γ, 0.5 ng/ml, R&D systems) for 12 hours. After maturation, supernatants were analyzed using an ELISA kit to quantify MIP-1α (R&D Systems, *Minneapolis*, MN) and TNF-α (eBioscience, San Diego, CA). The cells were evaluated for the cell marker expressions using a FACS CantoⅡ flow cytometer (BD Biosciences) after staining with specific antibodies; anti-CD11b-APC-Cy7 (BD Pharmingen, San Jose, CA), anti-CD206-PerCP-Cy5.5 (BioLegend, San Diego, CA), anti-CD68-APC (BioLegend) and anti-CD80-PE (eBioscience). To measure intracellular cytokine production, microglial cells were treated with the leukocyte activation cocktail with BD GolgiPlug (BD Biosciences) for 12 hours and with BD Cytofix/Cytoperm Fixation/Permeabilization kit (BD Biosciences), and then stained with the following antibodies; anti-MIP-1α-PE (eBioscience), anti-TNF-α-FITC (eBioscience), anti-interleukin-1β (IL-1β)-FITC (eBioscience), anti-IL-12p40/p70-APC (BD Pharmingen), anti-CD11b-APC-Cy7, and anti-CD206-PerCP-Cy5.5. The cells were finally analyzed with a flow cytometer.

### Neurotoxicity Assay

The mouse neuroblastoma Neuro-2A cell line (ATCC CCL-131) was maintained in MEM (Gibco) medium supplemented with 10% fetal calf serum (FBS, Gibco), non-essential amino acids (NEAA, Gibco) and 100 U/ml penicillium/streptomycin (Sigma-Aldrich). Neuro-2A cells were plated at a density of 4,000 per well in a 96-well cell culture plate (Essen Bioscience, Welwyn Garden City, UK). After 24 hours, FBS was added to a final concentration of 1% serum along with all-*trans* retinoic acid (atRA; Wako, Tokyo, Japan) to a final concentration of 10 μM in order to induce differentiation into neuronal cells. After removing the medium, the cells were incubated with H_2_O_2_ or microglial culture supernatant supplemented with 5 μM CellPlayer 96-well Kinetic Caspase-3/7 reagent (Essen Bioscience) and 150 μM Yo-Pro3 (Life Technologies, Gaithersburg, MD) for an additional 24 hours. Microglial culture supernatants were prepared by the following procedure. Firstly, microglia from newborn C57BL/6J mice were plated and pretreated with 0 or 5 μM DHE for 12 hours, followed by 5 ng/ml LPS and 0.5 ng/ml IFN-γ for 12 hours. Quantitative live cell imaging to assess apoptosis or cell-death was performed using an IncuCyte Zoom real-time imaging system (Essen Biosciences). Images were obtained every 3 hour at 20x magnification in phase contrast mode. The number of cells that had undergone apoptosis or cell-death was determined using the object counting algorithm.

### Evaluation of the anti-inflammatory activity of various types of cheese

Various types of cheese available in the marketplace were analyzed for their potential anti-inflammatory activity. The cheeses tested included fresh cheese (Mozzarella), various fermented cheeses (Camembert and Gorgonzola fermented using *P*. *candidum* and *P*. *roqueforti*, respectively) as well as semi-hard cheese (Gouda) or hard cheese (Grana Padano and Parmigiano Reggiano). The cheeses were freeze-dried, crushed and then treated with 5 volumes of *n*-hexane (Wako, Tokyo, Japan) to remove the triglyceride. Following *n*-hexane extraction, the remaining samples were treated with 2 volumes of methanol and then dried. Each sample was used at a concentration of 1 mg/ml for the anti-inflammatory assay.

### Extractions of camembert cheese

Camembert cheese was prepared by Koiwai Dairy Products Co. Ltd. (Tokyo, Japan) according to the standard manufacturing procedure with or without *P*. *candidum* fermentation. Briefly, sterilized milk was fermented with *Lactococcus lactis* and then treated with rennet. The aggregated curd was fermented with or without *P*. *candidum*. The products were freeze-dried, crushed and treated with 5 volumes of either *n*-hexane, chloroform, 50:50 chloroform/methanol mixture or methanol. Each extract was filtered through a glass filter unit (Whatman, GE Healthcare Japan, Tokyo, Japan), evaporated to dryness and used for anti-inflammatory evaluation.

### Fractionation of the methanol extract by HPLC

The methanol extract, prepared as described in the previous section, was dissolved in water. The extract was then applied to a Sep-Pak C18 cartridge column (Nihon Waters, Tokyo, Japan) before washing with water. Bound compounds were eluted with methanol and each fraction was fully dried and dissolved in a solution containing 90% water and 10% methanol before further separation by high performance liquid chromatography (HPLC, Shimadzu, Kyoto, Japan). Samples were separated on a 10 mm × 250 mm Develosil C30 column (Nomura Chemical Co. Ltd., Japan) at 25°C using a flow rate of 3.0 ml per minute. The separation protocol was as follows: ramping from 90% water / 10% methanol to 25% water / 75% methanol over 10 min and then to 100% methanol over 25 min, and holding for 25 min. Peaks at 265 nm, 285 nm and 320 nm were detected using SPD-M20A photodiode detection system (Shimadzu). The extract was fractionated into 12 samples (Fr.1-Fr.12). The peaks, with a retention time of 42.0–43.0 min, 44.0–45.0 min, 49.0–50.0 min and 52.0–55.0 min, were re-fractionated and used for the anti-inflammatory assay. The peak detected at 320 nm with a retention time of 49.0–50.0 min was purified for GC/MS analysis.

### GC/MS analysis

GC/MS was performed using an Agilent Technologies Network GC/MS system (7890 GC with 5975 mass detector) fitted with a HP-5MS capillary column (0.25 mm internal diameter, 0.25 μm film thickness, 30 m long, Agilent Technologies, Edinburgh, UK). The GC/MS method was as follows: initial temperature was 80°C with 1 min hold time, ramped to 280°C at 30°C per min and finally held at 280°C for 23 min. The total run time was 30.7 min. Helium was used as a carrier gas with a flow of 1.2 ml/min. Ionization was obtained by electron impact (electron energy, 70 eV). The temperature of the injection port and the transfer line was 250°C. The injection volume was 1 μl using a splitless injector.

### DHE production in fungi

*P*. *candidum* (PC12, PC NEIGE, PC22, PC HP6, PC SAM3, PC VS, PC ABL, Danisco Japan, Tokyo, Japan), *P*. *roqueforti* (PA, PJ, PV, Danisco), *Aspergillus awamori* (AOK1597 of black *Aspergillus* and AOK1598 of white *Aspergillus*, Akita-konno, Akita, Japan) were plated on 5% skimmed milk agar medium and cultured at 25°C for 5 days.

### HPLC analysis of extracts from fungi

The fungi were removed from the surface of the agar medium, and crushed in a mortar after lyophilization. The pieces of fungi were suspended in methanol containing 1 N KOH and β-sitosterol as an internal standard. The homogenates obtained by vortexing with glass beads were saponified at 80°C for 2 hours. Non-saponifiable lipid fractions were obtained as described previously [19] with some modification. Vehicle (10:9 chloroform:water) was added into the fraction (final ratio: approximately 1:1:0.9, v/v/v; chloroform:methanol:water) and vortexed for 20 min before centrifugation. The lower phase was dried in a stream of N_2_ at 40°C, re-dissolved in acetone, and filtered through a polytetrafluoroethylene membrane disc (Millipore, Bedford, MA). The non-saponifiable lipid fractions were analyzed by reversed phase HPLC on a Shimadzu Prominence HPLC system (Shimadzu, Kyoto, Japan) fitted with a fluorescence and UV-VIS detector. DHE and other sterols were separated using a Develosil C30-UG-3 (150 × 4.6 mm i.d., 3 μm) column (Nomura Chemical Co. Ltd., Japan) at 40°C. The mobile phase was acetonitrile-trifluoroacetic acid (100:0.1, v/v) with a flow rate of 1.0 ml per minute. Bound compounds were obtained by isocratic elution. DHE was detected with a fluorescence detector using an emission wavelength of 375 nm and excitation of 325 nm. Ergosterol and β-sitosterol were detected by absorbance at 210 nm. Sterol quantity was calculated from calibration curves using an internal standard of β-sitosterol.

### Statistical analysis

All results were expressed as the mean ± SEM. Unpaired *t*-test, one-way analysis of variance (ANOVA) (Dunnett) were used to compare the differences between groups. A two-tailed *P*-value<0.05 was accepted as significant for all tests. All statistical analyses were performed using the Ekuseru-Toukei 2012 program (Social Survey Research Information, Tokyo, Japan).

## Results

### Anti-inflammatory activity of a dairy product fermented with the *Penicillium* strain

**Fig 1:**
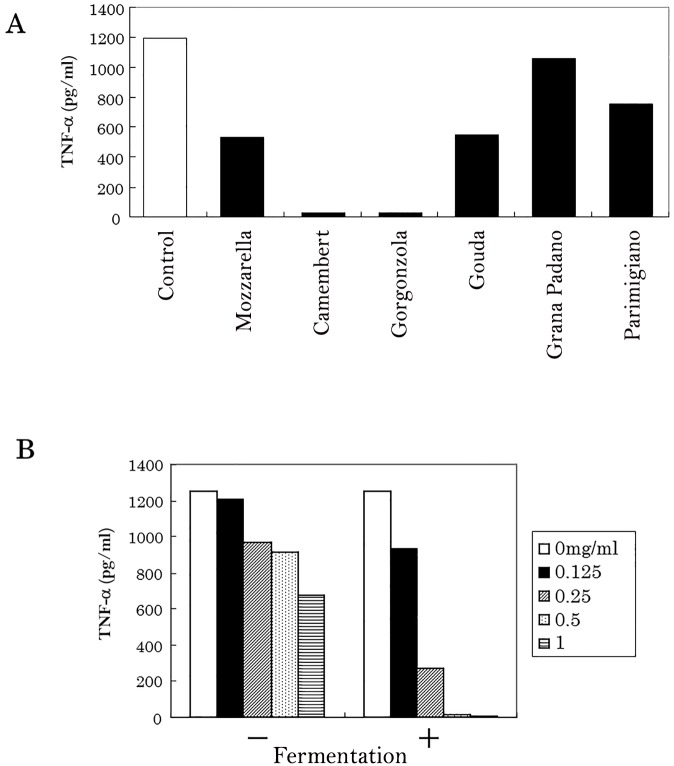
The effect of dairy products on the anti-inflammatory activity of microglial cells. (A) Isolated microglia were stimulated with 5 ng/ml LPS and 0.5 ng/ml of IFN-γ after pretreatment with an extract of each dairy product, and then TNF-α in the supernatant was quantified. Evaluation of the extraction and cytokine assay was repeated twice. (B) Effect of pretreatment with an extract of dairy product (with or without *P*. *candidum* fermentation) on the production of microglial TNF-α.

Extracts of Camembert cheese fermented with *P*. *candidum*, and Gorgonzola cheese fermented with *P*. *roqueforti*, was found to suppress microglial TNF-α production (Fig. 1A). None of the other types of cheese showed this biological activity. Moreover, the extract of dairy product fermented with *P*. *candidum* suppressed microglial TNF-α production in a concentration-dependent manner (Fig. 1B). Evaluation of the extraction and the cytokine assay was repeated twice.

### Identification of an anti-inflammatory ingredient in fermented dairy products

**Fig 2:**
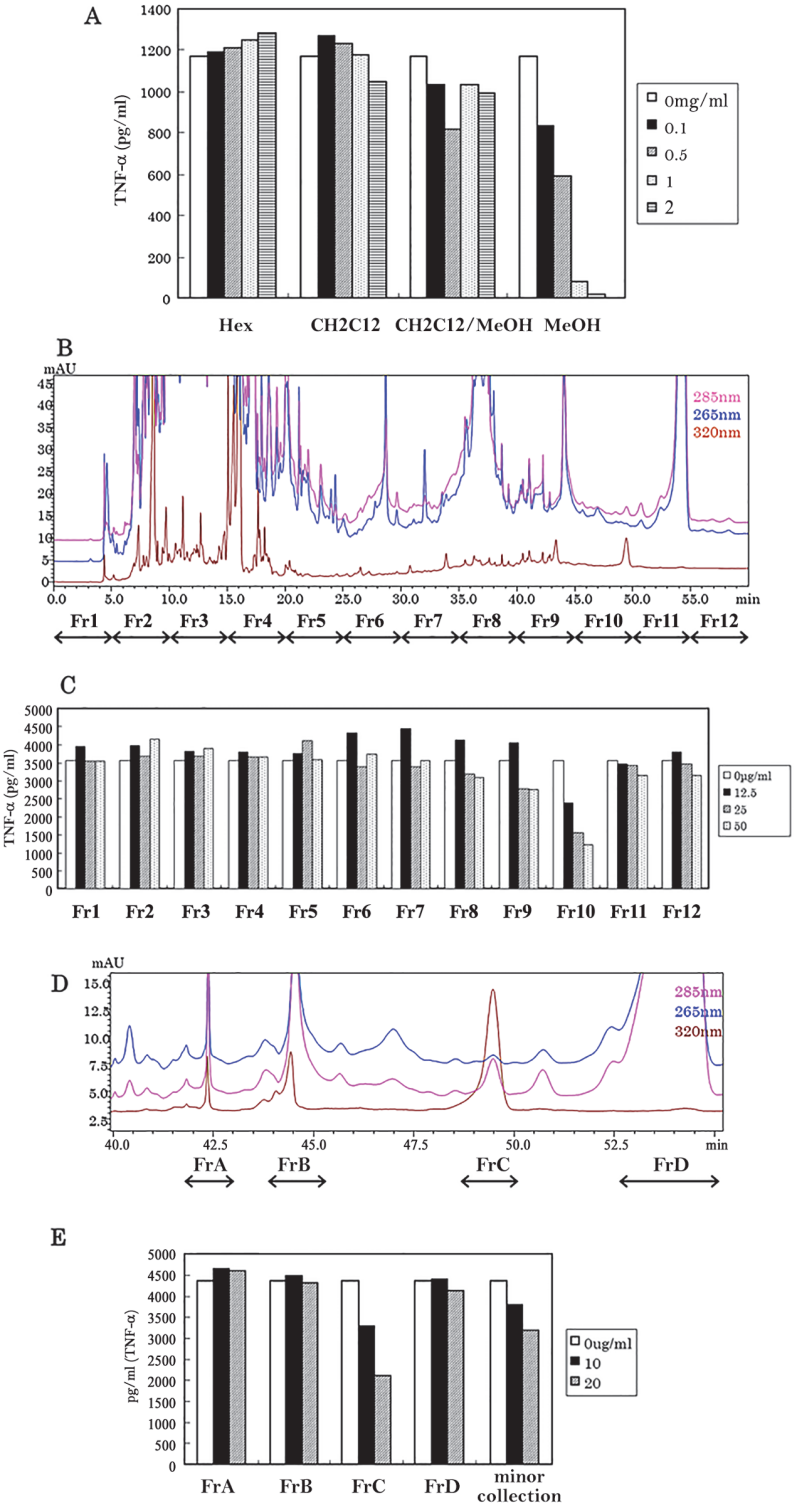
The effect of solvent extracts and fractionated samples on the anti-inflammatory activity of microglial cells. (A) Freeze dried camembert cheese was treated with either *n*-hexane, chloroform, chloroform/methanol mixture (1:1 by volume) or methanol. Each extract was evaporated to dryness before dissolving the residue in a suitable buffer. These solutions were then used in the microglial TNF-α production assay. Evaluation of the fractionation and cytokine assay was repeated twice. (B) Methanol extract of camembert cheese was fractionated into Fr1-Fr12 using a C30 column. (C) Effect of the pretreatment of Fr1-Fr12 on production of TNF-α by microglial cells. (D) Major peaks with a retention time of 40.0 min to 55.0 min were re-fractionated as FrA-FrD. (E) Effect of the pretreatment of FrA-FrD on the production of TNF-α by microglial cells.

Extracts of fermented Camembert cheese in methanol at a concentration of 0.5–2.0 mg/ml were found to suppress microglial TNF-α production in a concentration-dependent manner. By contrast, the extracts in *n*-hexane, chloroform and 50:50 chloroform:methanol mixture did not display any such biological activity (Fig. 2A). The weight of methanol extract was increased by the fermentation (data not shown). Methanol extracts were fractionated as shown by the UV spectrum patterns (Fig. 2B). Fr10, which had a retention time 45.0 to 50.0 min, displayed the most powerful suppression of TNF-α production in microglial cells. Moreover, the suppression of TNF-α production by Fr10 was concentration-dependent (Fig. 2C). Major peaks detected with a retention time from 45.0 to 55.0 were then purified (Fig. 2D) and used for the assay. FrC was found to potently suppress microglial TNF-α production in a concentration-dependent manner (Fig. 2E). Evaluation of the fractionation and cytokine assay was repeated twice.

### GC/MS analysis of FrC and DHE

**Fig 3:**
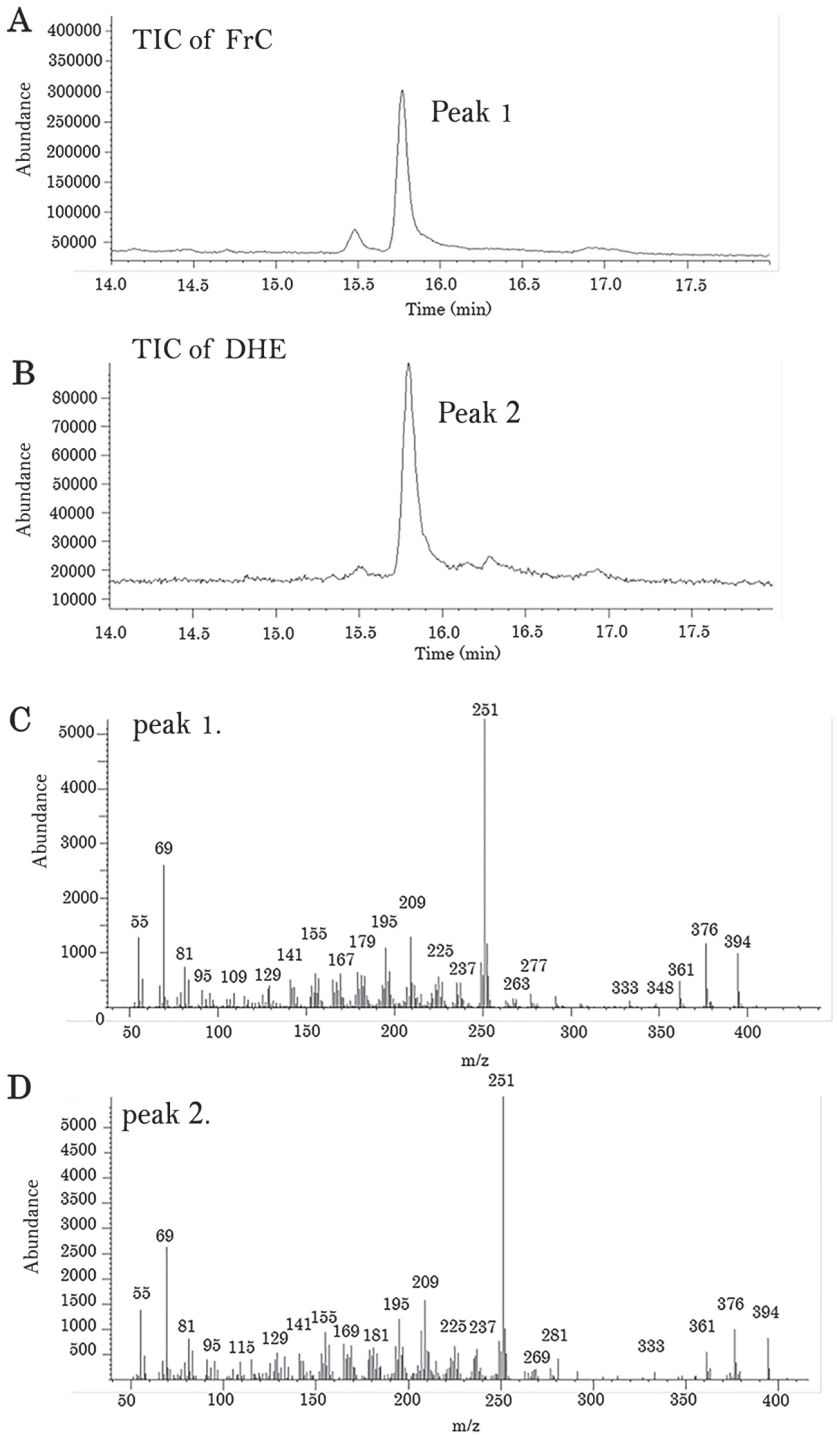
GC/MS analysis of fractionated peaks and DHE. GC/MS was performed using an Agilent Technologies Network GC/MS system (7890 GC plus a 5975 mass detector) with a HP-5MS capillary column. (A) GC/MS chromatogram of the fractionated peak as FrC. (B) GC/MS chromatogram of dehydroergosterol as a standard. (C) Mass spectrum of peak 1. (D) Mass spectrum of peak 2.

The enhanced microglial anti-inflammatory activity of FrC was analyzed by GC/MS. Both the chromatograph and mass spectrum of FrC (Fig. 3A and C) matched those obtained for DHE (Fig. 3B and D).

### Anti-inflammatory activity of DHE

**Fig 4:**
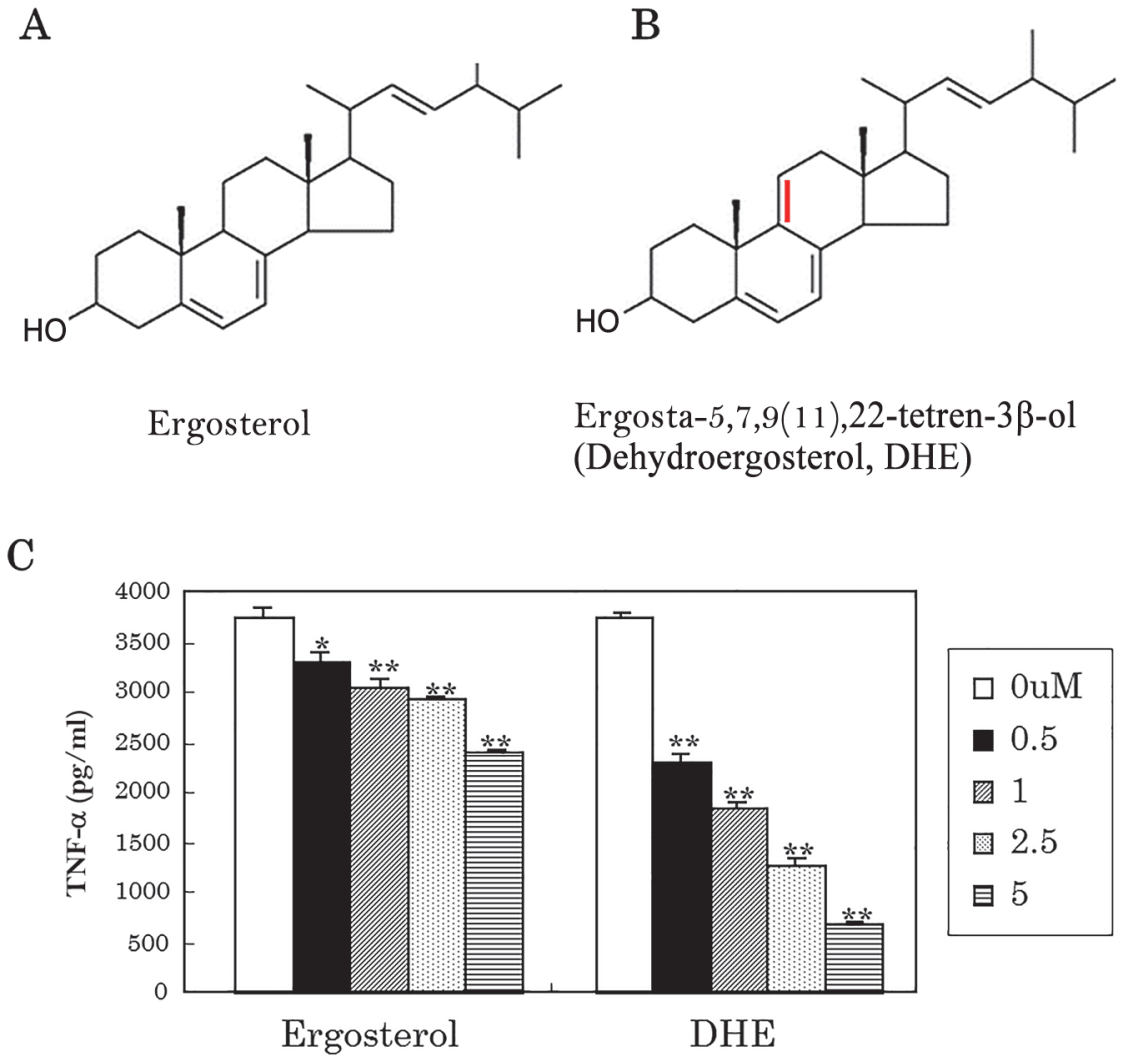
Anti-inflammatory activity of ergosterol and DHE on microglia. (A) Chemical structural formula of ergosterol. (B) Chemical structural formula of DHE. (C) Isolated microglial cells were stimulated with 5 ng/ml LPS and 0.5 ng/ml IFN-γ after pretreatment using ergosterol and DHE. The level of TNF-α in the supernatant was then measured by ELISA.

DHE (Fig. 4B) is an analogue of ergosterol (Fig. 4A) and contains a characteristic conjugated double bond system. DHE at a concentration of only 2.5 μM and 5 μM suppressed TNF-α production by more than 50%, whereas ergosterol was far less potent (Fig. 4C).

### DHE production by *P*. *candidum*

**Fig 5:**
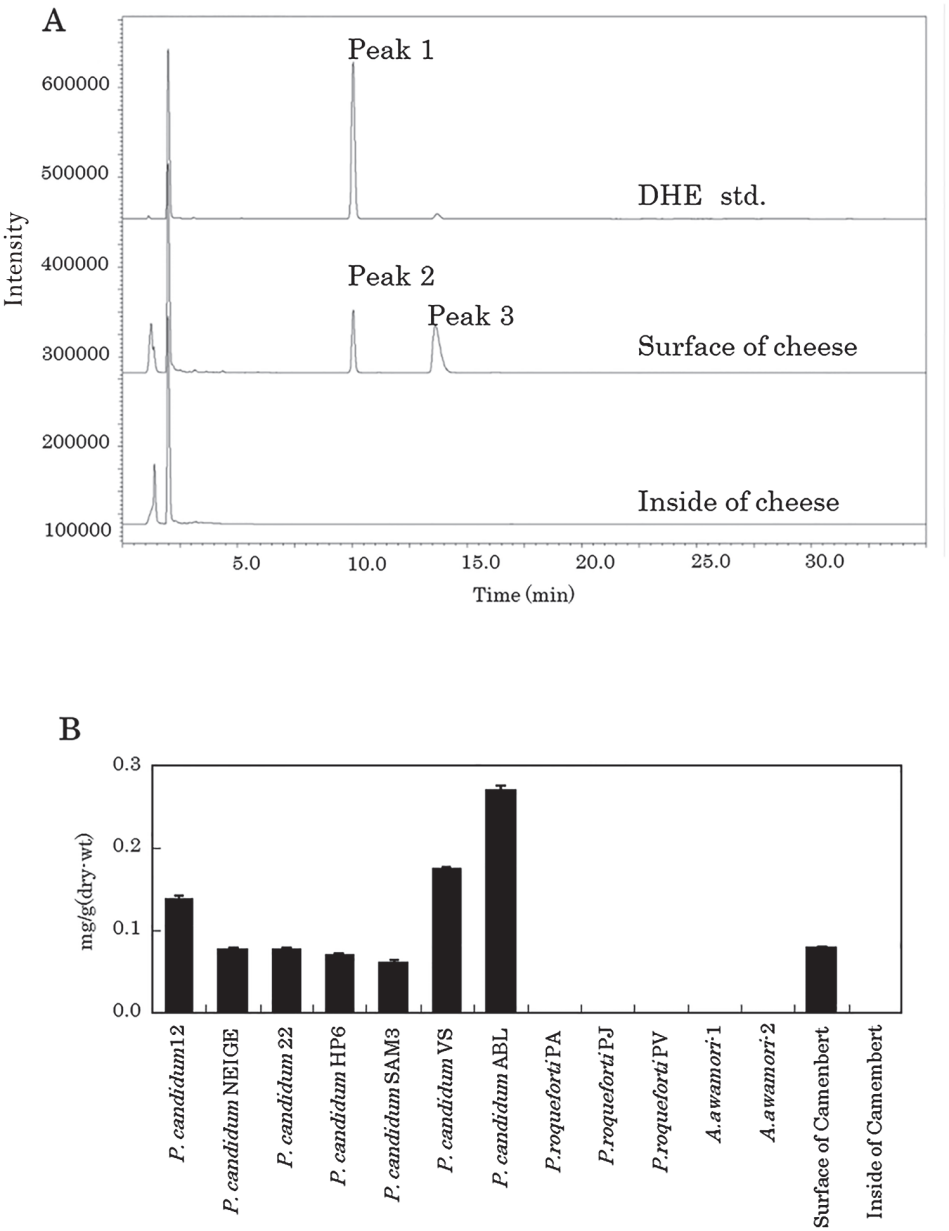
Quantification of DHE in a dairy product and fungal strain. (A) HPLC-fluorescence detection of DHE using samples taken from the surface and from the inside of a dairy product. Excitation as 325 nm and emission was 375 nm. (B) Quantification of DHE of *Penicillium* strains, *Aspergillus* strains and a dairy product.

DHE detected as peak 1 was also identified by fluorescent detection (emission wavelength of 375 nm and excitation of 325 nm) using a Develosil C30-UG-3 column and HPLC system (Fig. 5A). The surface of camembert cheese contains 0.08 mg/g of DHE (detected as peak 2), although this compound was undetectable in the interior of the cheese. Peak 3 in the HPLC trace corresponded to ergosterol (data not shown). *P*. *candidum* strains plated on 5% skimmed milk medium produced 0.05–0.28 mg/g of DHE. By contrast, DHE was undetectable in *P*. *roqueforti* and *Aspergillus* strains plated on the same medium. Moreover, as shown in Fig. 5B, DHE was abundant on the surface of dairy products fermented with *P*. *candidum*, but could not be detected on the inside of the cheese.

### Induction of microglia to an anti-inflammatory phenotype by treatment with DHE

**Fig 6:**
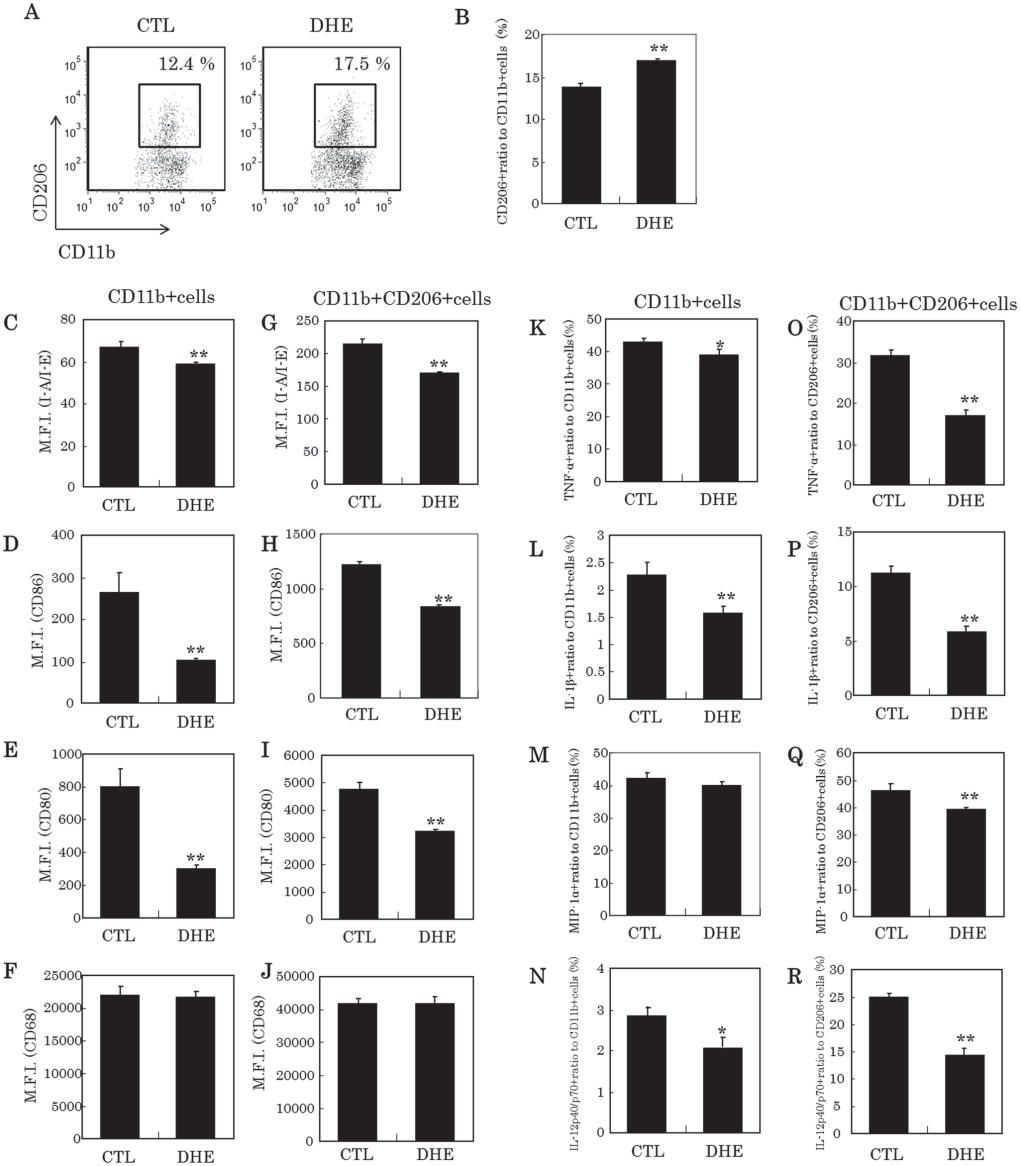
Characteristic of microglial cells treated with DHE using a flow cytometer. (A) Dot plot of microglia to gate CD11b-positive and CD206-positive cells treated either with or without DHE. (B) Ratio (%) of CD11b-positive and CD206-positive cells to CD11b-single positive cells. (C-F) Median fluorescent intensity of I-A/I-E, CD86, CD80 and CD68 in CD11b-positive cells. (G-J) Median fluorescent intensity of I-A/I-E, CD86, CD80 and CD68 in CD11b-positive and CD206-positive cells. K-N. Ratio (%) of TNF-α, IL-1β, MIP-1αin IL-12p40/p70-positive to CD11b-positive cells, respectively. O-R. Ratio (%) of TNF-α, IL-1β, MIP-1α and IL-12p40/p70-positive in CD11b-positive and CD206-positive cells, respectively.

The proportion of CD11b-positive and CD206-positive M2 type microglia significantly increased after treatment with DHE (Fig. 6A and B). Moreover, DHE significantly suppressed the expression of I-A/I-E, CD86 and CD80, but not CD68 (Fig. 6C-F). In particular, the expression of CD86 and CD80 in CD11b-positive microglia was markedly suppressed compared with CD11b-positive and CD206-positive microglia. DHE also significantly reduced the production of inflammatory cytokines and chemokines (TNF-α, IL-1β, MIP-1α, and IL-12p40/p70, Fig. 6K-N, respectively), which were highly suppressed in CD11b-positive and CD206-positive M2 type microglia (Fig. 6O-R).

### Protective effect of DHE for Neuro-2A *via* its anti-inflammatory activity

**Fig 7:**
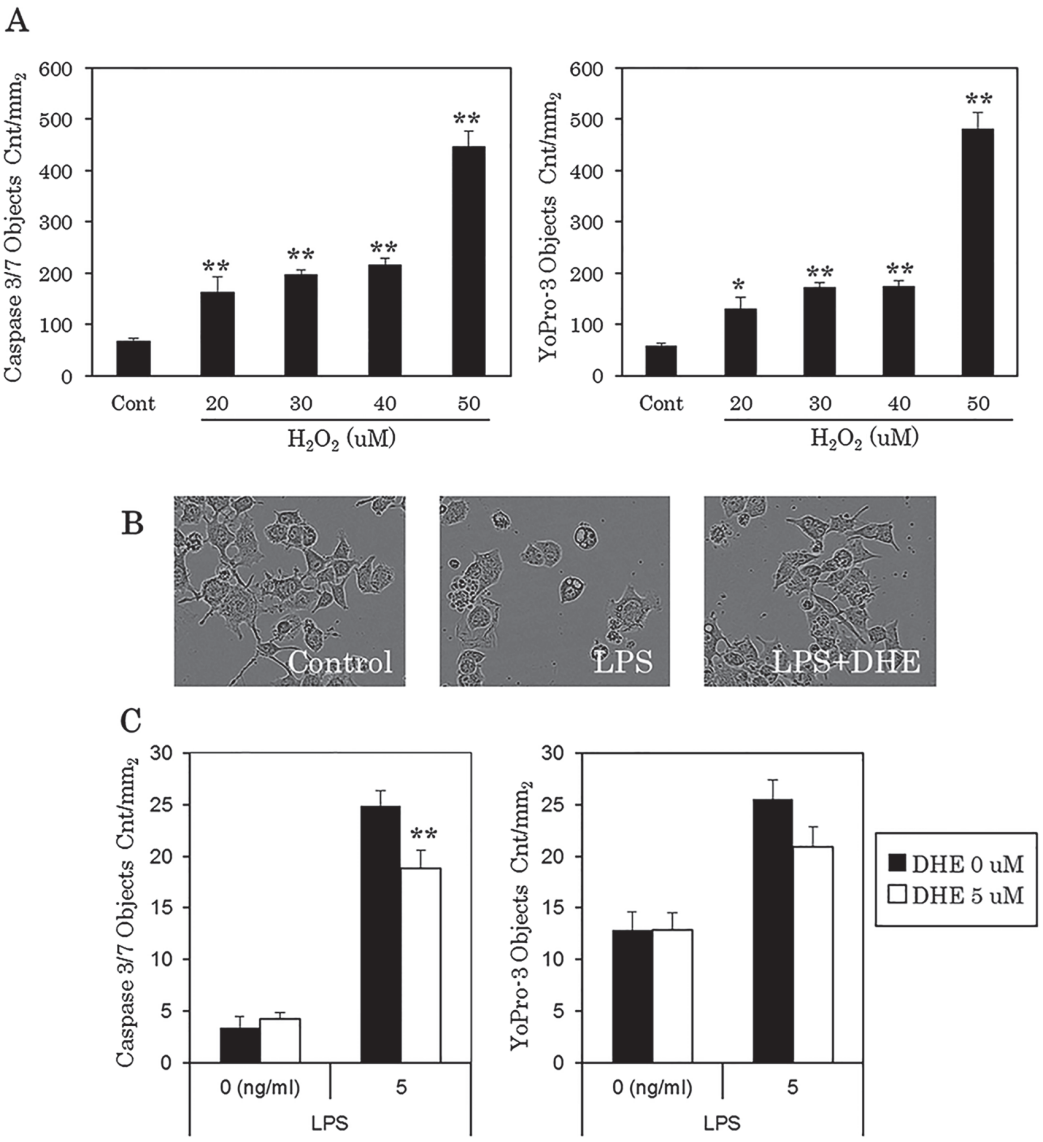
Neurotoxity assay of microglial culture supernatant treated with DHE. (A) Quantification of H_2_O_2_-induced apoptosis and cell-death in Neuro-2A cells. Left; caspase-3/7, Right; YoPro-3. (B) Representative phase contrast images of Neuro-2A cells. Left, image of untreated cells; Middle, image of cells treated with 5 ng/ml LPS only; Right, image of cells treated with 5 ng/ml LPS and 5 μM DHE. (C) Quantification of LPS-induced apoptosis and cell-death in Neuro-2A cells. Left; caspase-3/7, Right; YoPro-3.

H_2_O_2_ was used as a positive control for neurotoxicity induced cytotoxicity in the assay. Caspase-3/7 was used as an apoptosis marker and YoPro-3 as a cell-death marker after cell incubation for up to 24 hours. As shown in Fig. 7A, H_2_O_2_ at 20 to 50 uM significantly increased the level of both caspase-3/7 and YoPro-3. We used this assay to evaluate the potential anti-inflammatory activity of DHE for Neuro-2A cells. As illustrated in Fig. 7B, cells incubated with microglial supernatant pretreated with LPS for 24 hours resulted in alterations in cell morphology (e.g. shrinkage of synapse) compared with the control. However, these morphological changes were significantly reduced when the cells were incubated with microglial supernatant pretreated with both LPS and DHE. Moreover, the level of caspase-3/7 significantly decreased after treatment with this DHE supernatant (Fig. 7C).

## Discussion

Epidemiological studies suggested that ingestion of fermented dairy products could prevent a decline of recognition in the elderly [16–18]. Moreover, these epidemiological reports indicated that a specific, though unidentified, ingredient in dairy products elicits an anti-inflammatory or anti-oxidative effect in the brain for neuroprotection. In the present study, we identified DHE as a novel anti-inflammatory ingredient in a camembert-type cheese fermented with *P*. *candidum*.

Microglial cells are crucial for maintaining the environment in the central nervous system. Specifically, microglia are responsible for removing waste products such as Aβ and apoptotic cells by phagocytosis as well as promoting synapse formation by producing and releasing neurotrophic factors [3], [4]. However, in aged brain the continual presence of waste products, including Aβ, triggers chronic inflammation in microglia leading to their excessive activation [5–7]. Activated microglia produce huge amounts of cytokines, chemokines, ROS and NO that cause neuron cell death and finally a decline in recognition. Epidemiological studies suggest that prolonged use of nonsteroidal anti-inflammatory drugs (NSAIDs) can significantly reduce the risk of Alzheimer’s disease [13–15]. However, NSAIDs inhibit cyclooxygenase I thereby triggering side effects in the gastrointestinal tract, liver and kidney, which preclude the widespread use of these drugs for the prevention of Alzheimer’s. The possibility of regulating microglia to prevent dementia has attracted considerable attention, but an effective approach has not yet been developed. Therefore, in the present study, we screened for an ingredient in dairy products that can modulate the activity of microglia.

Initially, the anti-inflammatory activity of various dairy products was examined. Extracts of dairy products, especially those fermented with *Penicillum*, were found to display a potent anti-inflammatory effect on microglia. Moreover, we established that the anti-inflammatory effect is generated during the process of *Penicillium* fermentation. Repeated purification by HPLC, combined with evaluation using primary microglia, facilitated the identification of DHE as a novel component of the extract that enhances microglial anti-inflammatory activity. Although other fractions showed some anti-inflammatory activity, by far the most potent activity was detected in the DHE fraction.

DHE is a fluorescent substance that has been used as a probe for cholesterol trafficking [20], [21], but its physiological activity remains unclear. Indeed, this is the first study to report the anti-inflammatory activity of DHE. Despite their related chemical structures, the anti-inflammatory activity of DHE is markedly stronger than that of ergosterol. Inspection of these two chemical structures suggests that the conjugated double bond system is essential for anti-inflammatory activity. Ergosterol, which is known as provitamin D2, is particularly abundant in fungi as well as being present in bacteria and plants [22]. However, DHE, discovered in the present study, is found only in *P*. *candium*, and is not detectable in *Aspergillus* and *P*. *roqueforti*. We speculate that *P*. *candidum* might uniquely possess an enzyme to insert the double bond into ergosterol in the metabolic pathway. A specific strain of *P*. *candidum* produces 0.28 mg/g of DHE. Hence, dairy products containing tens of milligrams of DHE could be developed.

In addition, we established that DHE induces the transformation of CD11b-positive microglia into CD11b and CD206-positive microglia. CD11b-positive and CD206-positive monocytes, which are generally defined as M2 type, have an anti-inflammatory phenotype and wound-healing function when the tissue is damaged [23], [24]. DHE significantly suppresses the production of inflammatory cytokines and chemokines in M2 type microglia compared with CD11b-single positive microglia. Thus, DHE might induce microglia into a phenotype that blocks the onset of neurodegenerative diseases. This is because microglial cells in the brain of individuals with neurodegenerative disorders are induced into the M1 type, which produce large amounts of cytokines and chemokines, resulting in chronic inflammation [25–27]. DHE might have a potential not only to induce microglia but to change tissue macrophage into the M2-type in intestine, skin, and adipose tissues. In these tissues, chronic inflammations induced by tissue macrophage are closely related to dysfunction [28–30].

Treatment with DHE also reduces neurotoxicity from excessive microglial culture supernatant. Neuronal cell death, activation of caspase 3/7 and shrinkage of the synapse are all reduced by treatment with DHE, suggesting regulation of microglia by this means is beneficial for neurons. Moreover, because damage to the neuron by excessively activated microglia leads to cognitive decline, regulating microglia with DHE might also prevent impairment of cognitive ability [31–34].

In conclusion, we identified a novel functional compound, DHE, and it might be helpful in slowing down the deterioration in brain function by inducing a suitable microglial phenotype. However, the permeability of DHE through the blood- brain barrier was not assessed, so in the next step, we will evaluate the effect of DHE *in vivo* and permeability through the blood brain barrier. DHE is considered safe to consume and is present in various dairy products, such as camembert cheese. As such, DHE might be a valuable preventive tool for dementia.
